# Dietary Energy Density, Renal Function, and Progression of Chronic Kidney Disease

**DOI:** 10.1155/2016/2675345

**Published:** 2016-10-13

**Authors:** Mohammad Hossein Rouhani, Mojgan Mortazavi Najafabadi, Ahmad Esmaillzadeh, Awat Feizi, Leila Azadbakht

**Affiliations:** ^1^Food Security Research Center and Department of Community Nutrition, School of Nutrition and Food Science, Isfahan University of Medical Sciences, Isfahan, Iran; ^2^Department of Nephrology, Isfahan Kidney Diseases Research Center, Isfahan, Iran; ^3^Department of Community Nutrition, School of Nutritional Sciences and Dietetics, Tehran University of Medical Sciences, Tehran, Iran; ^4^Faculty of Epidemiology and Biostatistics, Isfahan University of Medical Sciences, Isfahan, Iran; ^5^Diabetes Research Center, Endocrinology and Metabolism Clinical Sciences Institute, Tehran University of Medical Sciences, Tehran, Iran

## Abstract

*Background*. There is evidence of the association between dietary energy density and chronic diseases. However, no report exists regarding the relation between DED and chronic kidney disease (CKD).* Objective*. To examine the association between dietary energy density (DED), renal function, and progression of chronic kidney disease (CKD).* Design*. Cross-sectional.* Setting*. Three nephrology clinics.* Subjects*. Two hundred twenty-one subjects with diagnosed CKD.* Main Outcome Measure*. Dietary intake of patients was assessed by a validated food frequency questionnaire. DED (in kcal/g) was calculated with the use of energy content and weight of solid foods and energy yielding beverages. Renal function was measured by blood urea nitrogen (BUN), serum creatinine (Cr), and estimated glomerular filtration rate (eGFR).* Results*. Patients in the first tertile of DED consumed more amounts of carbohydrate, dietary fiber, potassium, phosphorus, zinc, magnesium, calcium, folate, vitamin C, and vitamin B2. After adjusting for confounders, we could not find any significant trend for BUN and Cr across tertiles of DED. In multivariate model, an increased risk of being in the higher stage of CKD was found among those in the last tertile of DED (OR: 3.15; 95% CI: 1.30, 7.63; *P* = 0.01).* Conclusion*. We observed that lower DED was associated with better nutrient intake and lower risk of CKD progression.

## 1. Introduction

Chronic kidney disease (CKD) refers to a group of disorders in kidney structure and function [1]. Although CKD had been 27th cause of global total mortality in 1990, it moved up to 18th cause in 2010 [2]. Estimated global prevalence of CKD was 8–16% in 2013 [2]. Reports from a cross-sectional study published in 2009 showed that 18.9% of Iranian adults were diagnosed with CKD [3]. Dietary intake has an important role in prevention and treatment of CKD [4]. Although nutritional recommendations for patients with CKD have focused on limiting protein, sodium, potassium, and phosphorous intake [5], the importance of other aspects of diet (e.g., diet quality indices) should not be neglected. The association between diet quality indices and CKD was partially assessed previously. Findings of a research showed that nutritional quality of individuals with CKD was not adequate [6]. A recent study reported that there was a favorable association between a modified Alternative Healthy Eating Index score and progression of CKD [7]. Also, unhealthy dietary patterns were positively related to progression of CKD [8].

Dietary energy density, one of the most used diet quality indices, shows energy content of a given weight of diet [9]. There is a large body of evidence showing that DED is positively related to the risk of chronic diseases such as obesity [10], metabolic syndrome [11], and diabetes [12]. Also, DED was directly associated with chronic inflammation [13]. Moreover, previous study reported an increased oxidative stress among subjects who consumed high energy-dense meals [14]. A higher grade of inflammation and oxidative stress was observed among patients with CKD [15]. It was suggested that inflammation was directly related to CKD progression [16]. Therefore, we hypothesized that there was a relation between DED and biomarkers of renal function and CKD progression. As this association was not assessed previously, the aim of current study was to assess the relation between DED and markers of kidney function and progression of CKD.

## 2. Methods

### 2.1. Subjects

Two hundred twenty-one patients with diagnosed CKD were recruited for this study. Estimated glomerular filtration rate (eGFR) lower than 60 mL/min/1.73 m^2^ was determined as diagnostic criteria of CKD [17]. eGFR was calculated by Modification of Diet in Renal Disease (MDRD) method [18]. CKD was classified as stage 3 (30 ≤ eGFR ≤ 59 mL/min/1.73 m^2^), stage 4 (15 ≤ eGFR ≤ 29 mL/min/1.73 m^2^), and stage 5 (eGFR < 15 mL/min/1.73 m^2^) [17]. Other diseases (e.g., diabetes and nephrolithiasis) had been controlled by related specialist. Patients who signed written consent were recruited for this cross-sectional study. Therefore, we included patients with diagnosed CKD (eGFR < 60 mL/min/1.73 m^2^) who signed written consent form. Subjects who needed dialysis treatment were excluded.

### 2.2. Dietary Assessment and DED

An interviewer-administered food frequency questionnaire (FFQ) was used to assess dietary intake of patients during the previous year. The frequency of consumption of 168 food items was measured by foresaid FFQ. The validity and reliability of this semiquantitative FFQ were reported elsewhere [19]. Energy and nutrient content of consumed foods were analyzed by Nutritionist IV software (N-Squared Computing, Salem, OR). Daily energy intake <800 or >4200 kcal/d was considered as under- and overreporting, respectively. Under- and overreported data were excluded.

DED (in kcal/g) was calculated with the use of energy content and weight of solid foods and energy yielding beverages.

### 2.3. Biochemical Measures

After 12-hour overnight fasting, a blood sample was collected and then centrifuged at 3000 ×g for 10 min. Blood urea nitrogen (BUN) was measured by incubation of the blood sample with urease (intra-assay coefficient was 3.75 and interassay coefficient was 2.74). Serum creatinine (Cr) was determined by colorimetric reflectance spectrophotometry (intra-assay coefficient was 3.22 and interassay coefficient was 1.78). All kits were produced by Pars Azmoon Inc.

### 2.4. Other Variables

Demographic characteristics were assessed by oral questions. Subjects were asked regarding income, occupation, education, and region of residence to assess socioeconomic status. Weight was measured by digital scale to the nearest 0.1 kg. Height was assessed by self-reported measures. Participants were asked to report their sedentary time and activities to measure physical activity [20]. A trained assistance measured systolic and diastolic blood pressure by a standard mercury sphygmomanometer.

### 2.5. Statistical Analysis

Normality of the variables was tested using Kolmogorov-Smirnov test and histogram curve. We reported CKD stage, physical activity level (low, moderate, and high), marital status, sex ratio, and socioeconomic status (low, moderate, and high) as percentage frequency. Chi-square test was performed to detect difference in these variables. Continues variables (age, body mass index, biomarkers, and dietary intakes of nutrients) were presented as mean ± standard deviation. We used analysis of variance (ANOVA) to compared biochemical variables across the tertiles of DED. Analysis of covariance (ANCOVA) was performed to report energy-adjusted nutrient intakes across the tertiles of DED. Odds ratio and 95% confidence interval of the being in the higher stages of CKD were obtained using logistic regression. The risk was reported in crude and 2 adjusted models. The firs model was adjusted for age, physical activity, socioeconomic status, height, weight, and systolic and diastolic blood pressure. Further adjustment was performed for dietary intake of sodium, potassium, phosphorus, and animal protein per body weight in model 2. We considered *P* < 0.05 as significance level. Also, we used SPSS version 20 (IBM) to analyze this data.

## 3. Results

Demographic characteristics of patients with CKD across tertiles of DED are presented in Table 1. We did not observe any significant difference in demographic characteristics across tertiles of DED. The mean age of the patients in all three tertiles was more than 50 years. Most percentage of the subjects had middle socioeconomic status and low physical activity level. Also, most patients fell within stage 3 of CKD.

Nutrient intake of patients with CKD across tertiles of DED is reported in Table 2. The findings showed that patients in the first tertile of DED consumed more amounts of carbohydrate (*P* < 0.01), dietary fiber (*P* < 0.01), potassium (*P* < 0.01), phosphorus (*P* = 0.02), zinc (*P* = 0.02), magnesium (*P* < 0.01), calcium (*P* = 0.04), folate (*P* < 0.01), vitamin C (*P* < 0.01), and vitamin B2 (*P* < 0.01). In contrast, the intake of vitamin B3 in top tertile was significantly more than lower tertiles (*P* = 0.01).

Mean of renal function variables reported by tertiles of DED is presented in Table 3. We could not find any significant trend for BUN and Cr across tertiles of DED in crude and 3 adjusted models.

Odds ratios for being in the higher stage of CKD according to tertiles of DED are shown in Table 4. We did not observe a significant trend for risk of higher stage of CKD in crude model (*P* for trend = 0.18) and in model 1 (*P* for trend = 0.13). After adjusting for dietary confounders, an increased risk of being in the higher stage of CKD was found among those in the higher tertiles of DED (*P* for trend = 0.01).

## 4. Discussion

The results of the current study suggest that patients in the higher tertiles of DED had increased risk of being in the higher stage of CKD. To the best of our knowledge, this is the first study regarding the association between DED and risk of being in the higher stage of CKD. Furthermore, those in the lowest tertile of DED consumed higher amounts of different nutrients.

The results of the present study showed that patients in the first tertile of DED consumed more amounts of carbohydrate. We used energy-adjusted nutrient intake in the current study. Similar finding was observed in a large population-based study in which energy-adjusted nutrient intake was reported [21]. Also, results of an Iranian study conducted in young females supported our findings regarding carbohydrate [22]. Moreover, previous study reported that subjects who had low energy-dense diet consumed higher amounts of zinc, calcium, vitamin C, and vitamin B2 [23]. As patients in the first tertile of DED consumed more amounts of dietary fiber, it was suggested that large amounts of consumed carbohydrate were from fruits, vegetables, and whole grains. This hypothesis justifies our findings regarding higher intake of nutrients found in these foods (i.e., potassium, phosphorus, magnesium, folate, vitamin C, and vitamin B2).

Previous studies reported that high energy-dense diets may be associated with lower nutrient intake [22–24]. A large cross-sectional study reported that dietary fiber was related to reduction of inflammation and mortality among patients with CKD [25]. Therefore, beneficial effects of a low energy-dense diet on renal function may be mediated by dietary fiber and its effect on inflammation. Higher intake of dietary fiber may inhibit rapid digestion of carbohydrates which results in lower production of inflammatory mediators [26]. Recent evidence focused on higher consumption of dietary fiber in patients with CKD [27]. Intake of dietary fiber has been restricted in these patients because phosphorus content of foods rich in dietary fiber is high [5]. It should be noted that phosphorus is in phytate form in whole grains and legumes and its bioavailability is low [28]. Therefore, patients with CKD have been suggested to consume high amounts of dietary fiber found in whole grains and legumes.

An increased risk of being in a higher stage of CKD was observed among those in higher tertiles of DED in multivariable-adjusted model. Previous observational study reported that risk of incidence of CKD was higher among those who consumed more amounts of energy-dense, nutrient-poor sources of carbohydrate [29].

Our finding was adjusted for general (age, physical activity, socioeconomic status, height, weight, and blood pressure) and dietary (sodium, potassium, phosphorus, and animal protein intake) confounders. These confounders were selected according to the existing evidence. There was a significant association between age and outcomes of CKD [30]. Also, Cr might be affected by high physical activity level [31]. Socioeconomic status [32], anthropometric measurements [33], and blood pressure [34] were related to renal function and CKD. Moreover, kidney function may be affected by sodium intake [35] and animal protein [36, 37]. An elevated serum Cr was observed after meat consumption in a feeding study [38]. Moreover, a positive relation between meat intake and urea nitrogen was reported in clinical trials [39]. Also, nutritional recommendations for patients with CKD have focused on limiting potassium and phosphorous intake [5]. Therefore, we included all these confounder variables in our analysis.

Added sugars and fat are two means to achieve a high energy-dense diet [29]. Evidence showed that high sugar intake resulted in increased serum uric acid [40]. There is a positive association between elevated serum uric acid and hypertension which may lead to higher risk of renal damage [41]. On the other hand, observational studies reported a direct relation between high dietary fat intake and proteinuria [42]. It was suggested that the association between renal damage and dietary fat was mediated by inflammatory markers [42]. A positive relation between inflammatory marker (e.g., high sensitivity C-reactive protein) and saturated fatty acid was reported by an observational study [43]. Also, there is a significant positive association between high sensitivity C-reactive protein and proteinuria [44]. Therefore, inflammation can mediate the association between dietary fat (especially saturated fatty acid) and renal damage.

We should acknowledge a number of limitations. First, this was a cross-sectional study, and therefore we could not find a casual relationship. It is strongly recommended that future prospective cohort studies assess the association between DED and renal dysfunction. Second, we evaluate renal dysfunction by measuring BUN and creatinine. Future studies should measure other renal biochemical variables such as proteinuria. Third, we did not focus on a specific group of patients with CKD (e.g., diabetic nephropathy).

The strengths of present study are that we reported a new finding regarding the association between DED and chronic diseases. As mentioned, most previous studies focused on the relation between DED and cardiovascular diseases, diabetes and obesity. Therefore, this is the first study regarding the association between DED and kidney disease.

In conclusion, we observed that lower DED was associated with better nutrient intake and lower risk of CKD progression.

## Figures and Tables

**Table 1:** Demographic characteristics of patients with chronic kidney disease across tertiles of dietary energy density.

Variables	Tertiles of dietary energy density			*P* ^2^
	T1 (≤0.7 kcal/g) (*n* = 74)	T2 (0.8–0.9 kcal/g) (*n* = 73)	T3 >0.9 kcal/g (*n* = 74)	
Age (year)	56.53 ± 14.70^1^	58.04 ± 13.82	55.16 ± 17.02	0.52
Male (%)	64.9	67.1	75.7	0.32
Height (cm)	168.26 ± 8.81	167.16 ± 7.70	169.35 ± 9.76	0.32
BMI (kg/m^2^)	25.49 ± 3.66	25.96 ± 4.21	26.08 ± 5.10	0.68
Physical activity (%)				
Low	60.8	63.0	62.2	
Moderate	36.5	34.2	33.8	
High	2.7	2.7	4.1	0.98
Socioeconomic status (%)				
Low	9.5	20.5	24.3	
Middle	74.3	63.0	59.5	
High	16.2	16.4	16.2	0.18
CKD stage (%)				
Stage 3	67.6	74.0	59.5	
Stage 4	29.7	24.7	40.5	
Stage 5	2.7	1.4	0.0	0.18

BMI: body mass index; CKD: chronic kidney disease.

^1^Mean ± SD.

^2^Calculated by Chi-square and analysis of variance for qualitative and quantitative variables, respectively.

**Table 2:** Nutrient intake of patients with chronic kidney disease across tertiles of dietary energy density.

Variables	Tertiles of dietary energy density			*P* ^2^
	T1 (≤0.7 kcal/g) (*n* = 74)	T2 (0.8–0.9 kcal/g) (*n* = 73)	T3 >0.9 kcal/g (*n* = 74)	
Carbohydrate (g)	198.31 ± 61.68^1^	171.26 ± 67.22	133.14 ± 80.15	<0.01
Protein (g)	30.05 ± 11.34	29.36 ± 8.61	30.58 ± 13.85	0.81
Fat (g)	24.41 ± 7.99	24.01 ± 8.61	25.58 ± 13.85	0.27
Saturated fatty acid (g)	7.75 ± 7.99	7.40 ± 3.82	8.84 ± 4.79	0.10
Cholesterol (g)	147.56 ± 45.71	149.02 ± 42.89	153.40 ± 52.46	0.74
Dietary fiber (g)	15.07 ± 4.86	14.62 ± 6.92	13.68 ± 3.21	<0.01
Sodium (mg)	2425.38 ± 340.38	2334.14 ± 253.63	2439.59 ± 505.21	0.19
Potassium (mg)	1909.82 ± 853.64	1488.08 ± 444.86	1101.94 ± 531.74	<0.01
Phosphorus (mg)	770.78 ± 287.62	676.95 ± 196.15	651.99 ± 317.48	0.02
Zinc (mg)	6.42 ± 1.68	5.91 ± 1.35	5.67 ± 1.74	0.02
Magnesium (mg)	214.48 ± 55.43	185.00 ± 33.29	158.51 ± 42.65	<0.01
Calcium (mg)	910.35 ± 358.81	818.68 ± 214.23	790.68 ± 295.39	0.04
Folate (*µ*g)	264.92 ± 110.96	210.29 ± 46.64	184.66 ± 97.15	<0.01
Vitamin E (mg)	13.39 ± 6.67	12.78 ± 6.37	12.84 ± 5.79	0.81
Vitamin C (mg)	110.42 ± 103.95	88.78 ± 44.65	70.79 ± 39.80	<0.01
Vitamin B1 (mg)	1.49 ± 0.17	1.49 ± 16	1.51 ± 0.34	0.80
Vitamin B2 (mg)	1.46 ± 0.46	1.25 ± 0.28	1.19 ± 0.40	<0.01
Vitamin B3 (mg)	15.12 ± 2.27	16.07 ± 3.11	16.80 ± 4.67	0.01

^1^All values are mean ± SD and adjusted for total energy intake.

^2^Calculated by multivariate analysis of variance.

**Table 3:** Mean of renal function variables reported by tertiles of dietary energy density among patients with chronic kidney disease.

Variables	Tertiles of dietary energy density			*P* ^2^
	T1 (≤0.7 kcal/g) (*n* = 74)	T2 (0.8–0.9 kcal/g) (*n* = 73)	T3 (>0.9 kcal/g) (*n* = 74)	
BUN (mg/dL)				
Crude	26.72 ± 12.90^1^	28.15 ± 13.50	29.12 ± 12.90	0.45
Model 1	28.97 ± 13.33	30.98 ± 13.32	31.77 ± 13.33	0.42
Model 2	28.89 ± 13.07	30.75 ± 13.06	32.06 ± 13.16	0.42
Model 3	28.95 ± 13.50	30.72 ± 12.98	32.04 ± 13.93	0.42
Creatinine (mg/dL)				
Crude	1.85 ± 1.40	1.87 ± 1.36	2.02 ± 1.37	0.20
Model 1^3^	1.97 ± 0.69	1.98 ± 0.68	2.12 ± 0.69	0.38
Model 2^4^	1.97 ± 0.67	1.97 ± 0.72	2.12 ± 0.67	0.38
Model 3^5^	1.97 ± 0.76	1.98 ± 0.72	2.13 ± 0.78	0.40

BUN: blood urea nitrogen.

^1^Mean ± SD.

^2^Calculated by multivariate analysis of variance (in crude model) and multivariate analysis of covariance (in adjusted model).

^3^Model 1: adjusted for age, sex, physical activity, and socioeconomic status.

^4^Model 2: model 1 + systolic and diastolic blood pressure.

^5^Model 3: model 2 + weight, height, sodium, potassium, phosphorus, and animal protein intake per body weight.

**Table 4:** Odds ratios and 95% confidence intervals for being in the higher stage of chronic kidney disease according to tertiles of dietary energy density among patients with chronic kidney disease.

Models	Tertiles of dietary energy density			*P* for trend^1^
	T1 (≤0.7 kcal/g) (*n* = 74)	T2 (0.8–0.9 kcal/g) (*n* = 73)	T3 >0.9 kcal/g (*n* = 74)	
Crude	1	0.73 (0.36, 1.50)	1.42 (0.72, 2.48)	0.18
Model 1^2^	1	0.71 (0.34, 1.51)	1.52 (0.74, 3.12)	0.13
Model 2^3^	1	3.48 (1.55, 7.79)	3.15 (1.30, 7.63)	0.01

^1^ *P* value was calculated by logistic regression.

^2^Model 1: adjusted for age, physical activity, socioeconomic status, height, weight, and systolic and diastolic blood pressure.

^3^Model 2: model 1 + sodium, potassium, phosphorus, and animal protein intake per body weight.

## Abbreviations

ANOVA: Analysis of variance
BUN: Blood urea nitrogen
CKD: Chronic kidney disease
Cr: Creatinine
DED: Dietary energy density
eGFR: Estimated glomerular filtration rate
FFQ: Food frequency questionnaire.

## Additional Points

*Practical Application. *Dietitians should recommend a low energy-dense diet to patients with CKD to delay progression of CKD.
